# Optimal Timing and Duration of Induction Therapy for HIV-1 Infection

**DOI:** 10.1371/journal.pcbi.0030133

**Published:** 2007-07-13

**Authors:** Marcel E Curlin, Shyamala Iyer, John E Mittler

**Affiliations:** 1 Department of Medicine, University of Washington, Seattle, Washington, United States of America; 2 Department of Microbiology, University of Washington, Seattle, Washington, United States of America; Imperial College London, United Kingdom

## Abstract

The tradeoff between the need to suppress drug-resistant viruses and the problem of treatment toxicity has led to the development of various drug-sparing HIV-1 treatment strategies. Here we use a stochastic simulation model for viral dynamics to investigate how the timing and duration of the induction phase of induction–maintenance therapies might be optimized. Our model suggests that under a variety of biologically plausible conditions, 6–10 mo of induction therapy are needed to achieve durable suppression and maximize the probability of eradicating viruses resistant to the maintenance regimen. For induction regimens of more limited duration, a *delayed*-induction or -intensification period initiated sometime after the start of maintenance therapy appears to be optimal. The optimal delay length depends on the fitness of resistant viruses and the rate at which target-cell populations recover after therapy is initiated. These observations have implications for both the timing and the kinds of drugs selected for induction–maintenance and therapy-intensification strategies.

## Author Summary

Clinicians treating HIV infection must balance the need to suppress viral replication against the harmful side effects and significant cost of antiretroviral therapy. Inadequate therapy often results in the emergence of resistant viruses and treatment failure. These difficulties are especially acute in resource-poor settings, where antiretroviral agents are limited. This has prompted an interest in induction–maintenance (IM) treatment strategies, in which brief intensive therapy is used to reduce host viral levels. Induction is followed by a simplified and more easily tolerated maintenance regimen. IM approaches remain an unproven concept in HIV therapy. We have developed a mathematical model to simulate clinical responses to antiretroviral drug therapy. We account for latent infection, partial drug efficacy, cross-resistance, viral recombination, and other factors. This model accurately reflects expected outcomes under single, double, and standard three-drug antiretroviral therapy. When applied to IM therapy, we find that (1) IM is expected to be successful beyond 3 y under a variety of conditions; (2) short-term induction therapy is optimally started several days to weeks after the start of maintenance; and (3) IM therapy may eradicate some preexisting drug-resistant viral strains from the host. Our simulations may help develop new treatment strategies and optimize future clinical trials.

## Introduction

The failure of antiretroviral therapies to completely suppress viral replication in some patients represents a major difficulty in the management of HIV infection. In therapy-naive patients without clinically apparent resistance mutations, triple-drug therapy with two nucleoside–analog reverse transcriptase inhibitors and a protease inhibitor or a non-nucleoside reverse transcriptase inhibitor is standard [1]. In these patients, treatment success rates, defined as viral load <50 copies/ml at 48 wk, range from 70% to 80%–85% (reviewed in [2]). However, in patients with previous regimen failure requiring salvage therapy, response rates are usually considerably lower [3–5], and it is frequently not possible to assemble a three-drug regimen with uncompromised activity against all viral strains present. In these individuals, treatment failure often occurs after an initial period of response to a new regimen, and is usually associated with the appearance of multiply drug-resistant viral strains. This has led to attempts to treat highly experienced patients with various deep salvage regimens consisting of four, five, or six individual drugs [6–11]. These patients are particularly vulnerable to the many drug interactions [12] (also reviewed in [13]) and adverse metabolic, hematologic, neurologic, cardiovascular, and gastrointestinal side effects that complicate HIV therapy and seriously undermine the success of clinical management [14–20] (also reviewed in [21]).

The need to minimize drug resistance while reducing treatment-related toxicities has engendered an interest in induction–maintenance (IM) strategies, in which a period of intensified antiretroviral therapy (induction phase) is followed by a simplified long-term regimen (maintenance phase) [22–25]. Most such trials have yielded higher failure rates in the treatment group than in controls receiving conventional therapy. Failure typically occurs during maintenance therapy, and has been attributed to poor regimen adherence [25] and recrudescence of resistance mutations present before institution of induction therapy [23]. One weakness of existing studies has been that induction therapy consisted of standard three-drug antiretroviral therapy (ART) regimens in common clinical use at the time of the study, under conditions now recognized to permit subclinical viral replication [26,27]. Moreover, in these early studies, the induction phase only lasted between 3 to 6 mo, which may be insufficient. However, two recent studies have shown the apparent effectiveness of induction therapy for 48 wk followed by maintenance therapy with atazanavir [28] or lopinvir/ritonavir [29,30], and this has led to new optimism concerning IM approaches.

We have hypothesized that a longer period of a highly suppressive induction therapy that is appropriately timed relative to the start of maintenance therapy may allow minority resistant variants to decay below a stochastic extinction threshold, allowing for successful long-term treatment with simpler and better-tolerated regimens. To explore this hypothesis quantitatively, we constructed a detailed computer simulation model of the dynamics of sensitive and resistant viruses during a hypothetical IM regimen. We show that the timing and duration of induction therapy relative to maintenance therapy can affect the probability that viruses resistant to the maintenance regimen will be eradicated in ways that are somewhat counterintuitive. Under biologically plausible conditions, we find that 6–10 mo of induction therapy are required to maximize the probability of eradicating these resistant viruses. For shorter induction periods, we find that it is optimal to use a “delayed-induction” regimen administered several days to weeks after the start of the intended long-term maintenance therapy.

## Results

### Overview of the Model and Parameters

The model consists of CD4^+^ target cells, free viruses, and three types of infected cells: short-lived infected cells with *t*_1/2_ of ∼1 d, moderately long-lived infected cells with *t*_1/2_ of ∼2.5 wk, and long-lived infected cells or “latently” infected cells with *t*_1/2_ of ∼3.5 y (Figure 1A). The model includes four possible mutations that confer resistance to three antiretroviral drugs; mutations 1 and 2 each confer partial resistance to drug I, whereas mutations 3 and 4 confer a high level of resistance to drugs II and III, respectively (Figure 1B). Our model allows viral recombination, and includes the effects of partial drug efficacy, incomplete viral resistance, and cross-resistance between drugs II and III. Drug-resistant viruses can infect moderately long-lived and latently infected cells, allowing for the formation of latent drug-resistant viral reservoirs. Because the model assumes finite population sizes, the various viral genotypes may fall below a threshold for extinction. Since extinction is a chance event, we used random, stochastic modeling terms to model the rate of change of free viruses and infected cell populations that are near the extinction threshold.

**Figure 1:**
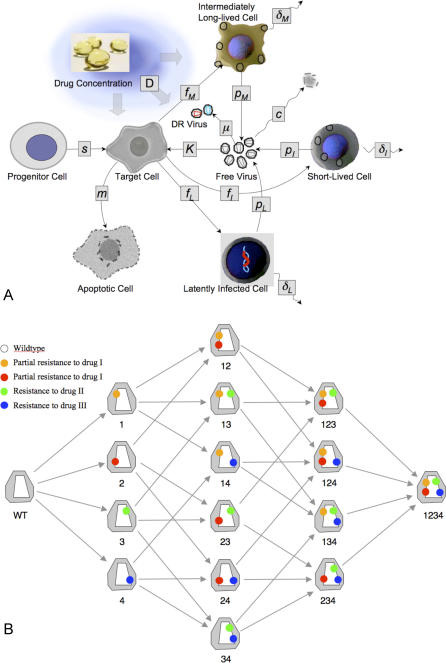
Overview of Cell Populations (A) and Mutations Responsible for Resistance (B) Mutation accumulation was modeled as a sequential process in which each genotype can acquire a single additional mutation in any given time-step (0.002 d in our simulations). In a single time step, *V*_1_, for example, could mutate to *V*_12_, *V*_13_, or *V*_14_, but not to *V*_123_. The model also allows for recombinational steps (see text), which are not depicted here.

### Viral Dynamics during Untreated Early and Chronic Infection

In the absence of therapy, viral load rises to a peak of approximately 10^6^ virions/ml by day 25, then falls to an equilibrium of ∼10^5^ virions/ml by day 100. Target-cell populations decrease during acute viremia, then recover somewhat as viral load falls to its steady state. (Analytical formulas for the steady-state concentrations of infected cells and free virus under a model very similar to the one here can be found in [31–37].) As observed in [31–37], our model assumes that resistant viruses have lower fitness in the absence of drug. With our conservative parameter choices, viruses with one, two, and three drug-resistance mutations are generally present at frequencies of 10^−3^, 10^−6^, and 10^−9^, respectively, during the period of acute primary infection, whereas viruses with four drug-resistance mutations are generally absent (Figure 2A). Thereafter, the frequency of mutants and latently infected cells (unpublished data) increase slowly to equilibrium. To account for this increase in our simulations, we allowed viral populations to equilibrate over a 4,000-d period (>10 y) before initiating therapy. With less conservative parameter choices, viruses with three resistance mutations will not generally preexist. In this case, the qualitative results described below can be duplicated with less intensive drug therapies.

**Figure 2:**
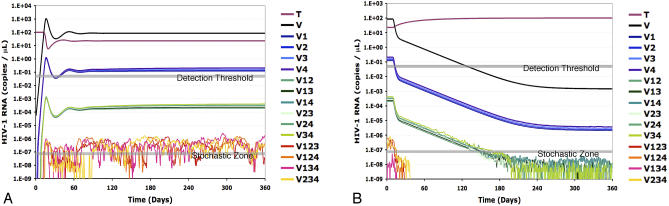
Simulations of Viral Dynamics (A) Dynamics in the absence of therapy. (B) Decline in viral load during potent triple-drug combination therapy. Maintenance and inducer drugs are provided for 360 d starting on day 0. Dark blue line, target cells; black line, WT virus; blue-green lines, single mutants; orange lines, double mutants; red lines, triple mutants. Viral populations that are above the threshold for stochastic effects (dark gray line) may fluctuate if the corresponding infected cell populations are below the cutoff for stochastic effects. After the initiation of therapy, WT virus declines with appropriate first-, second-, and third-order kinetics. Viruses with a single mutation decline to near steady-state levels above the extinction threshold. Viruses with two resistance mutations approach the extinction threshold, but are not entirely eliminated by day 300. Triple mutants are generally extinct by day 40.

### Viral Dynamics during Conventional ART

After initiation of conventional triple-drug therapy, the viral load decays at a rate of 0.6/d (first phase decay) for ∼10 d, then at 0.04/d (second phase decay), until HIV-1 RNA falls below the detection limit of 50 RNA copies (25 virions) per ml of plasma around day 120 (Figure 2B). A population of latently infected cells is assumed to contribute a third phase of decay beginning around day 200, during which virus decays at a rate of 0.00052/d. Viral loads during the third phase are on the order of 1.0/ml [40]. Model behavior during primary infection, chronic disease, and ART has been designed to match experimental viral dynamics [38–40]. The minority populations of resistant mutants form a reservoir of drug-resistant viruses that can fuel viral rebound if therapy is prematurely reduced or withdrawn. As expected, at low population densities under conditions prevailing during induction therapy, the appearance and loss of drug-resistant populations behave as random, stochastic processes.

### IM Therapy: Effect of Timing and Duration of Induction Therapy on the Probability of Eradicating Viruses Resistant to the Maintenance Regimen

We have used this model to investigate two questions about IM therapies. (1) How long should the induction phase be in order to eradicate viruses resistant to the drugs in the maintenance regimen? (2) What is the optimal timing of induction therapy relative to maintenance therapy? Could IM therapies be improved, for example, if the agents that were unique to the induction regimen were started before starting the maintenance drugs? In the simulations below, the maintenance regimen consists of drugs I and II, while drug III is applied only during induction therapy (Figure 3). We define “success” as achieving and maintaining a fully suppressed circulating free virus population for a period of at least 3 y after the end of induction therapy.

**Figure 3:**
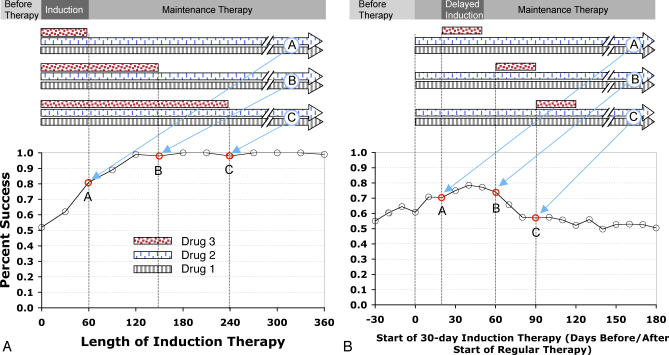
Schematic Illustrating Treatment Strategies Investigated in This Study (A) Effect of progressively longer induction regimens (circles A–C) on the likelihood of successfully eradicating viruses resistant to maintenance therapy under our canonical parameter set. (B) Effect of altering the timing of induction therapy (circles A–C) relative to maintenance therapy on the likelihood of successful therapy. *x*-Axis indicates duration of induction therapy in days (A), or interval between start of the induction and maintenance therapies, in days (B). Maintenance therapy is assumed to start on day 0. *y*-Axis indicates percentage of simulations in which viral load remained undetectable for at least 3 y after ending induction therapy.

Figure 3A–3B gives typical results; Figure 3A shows how the probability of success varies with the length of the induction phase. In this simulation, the percentage of success increased dramatically as the length of the induction therapy was increased to ∼120 d, and increased more gradually between 120 and 180 d. Further increases in the length of the induction phase beyond 180 d had little effect with these parameters. Figure 3B shows a typical simulation in which the timing of induction therapy was altered. In these simulations, a 30-d course of therapy intensification was started before maintenance therapy (start days −30 to −10), at the same time as maintenance therapy (start day 0), or after drugs unique to the maintenance therapy were started (start days 10 and higher). In the latter case, we refer to the period of intensified therapy as a “delayed-induction” therapy. Interestingly, we note that for induction therapies of limited duration, the highest success rates occurred with delayed-induction therapy initiated ∼40 d after the start of maintenance therapy.

Delayed-induction therapy (also referred to as delayed-intensification or booster therapy) results in higher eradication rates because drug-resistant viral populations are predicted to decline transiently after the start of maintenance therapy [41–43]. This decline occurs because resistant viruses, which are assumed to be less fit than sensitive viruses [31–37], are no longer created via mutation once drug therapy interrupts viral replication within the drug-sensitive population. Drug-resistant populations do not increase until target-cell populations increase enough to offset their intrinsic growth rate disadvantage. Specifically suppressing replication of resistant viruses with additional drugs when this population is reduced in size maximizes the net impact of induction therapy. This result can be shown analytically using a simple one-infected cell, one-resistant virus, deterministic version of this model in which wild-type (WT) virus is completely sensitive to drug, and resistant virus is completely resistant to drug (Figure 4A and 4B). With these simplifications, Nowak et al. [41] have shown that the dynamics of resistant virus after therapy is approximately

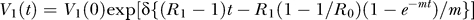
where *V*_1_(0) is the density of the resistant virus at the time that therapy is initiated, *m* is the turnover rate of target cells at steady state, *δ* is the death rate of infected cells, *R*_0_ = *psk*/*cδm*, and *R*_1_ = *psk*_1_/*cδm. R*_0_ and *R*_1_ are the basic reproductive numbers (i.e., the mean number of new cells infected from a single infected cell in a newly infected host who is not being treated) for WT and resistant viruses [41]. For *t* ≪ 1 / *m* and 0 < *R*_1_ < *R*_0_, the second term inside the curly brackets is large compared with the first, leading to transient declines in *V*_1_. As *t* becomes large compared with 1/*m*, this second term approaches *R*_1_ (1 − *R*_0_) / *m*, whereas the first term continues to increase linearly with *t*, allowing for eventual increases in *V*_1_. Setting the derivative of *V*_1_(*t*) equal to zero, it is straightforward to show that *V*_1_ reaches a nadir at

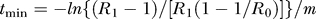



**Figure 4:**
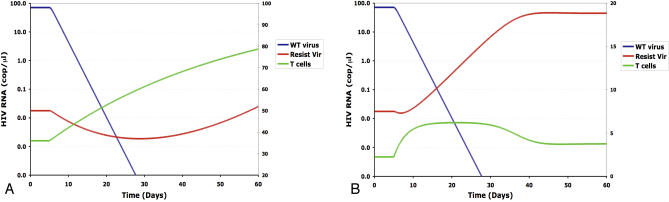
Deterministic Model of the Dynamics of Resistant Viruses under the One-Drug, One-Mutant, One-Cell Version of Our Target-Cell Model (A) Slow turnover rates for CD4^+^ target cells (*m* = 0.02, *k* = 0.0005). (B) Rapid turnover rates for CD4^+^ target cells (*m* = 0.32, *k* = 0.008). Here *m* and *k* were increased proportionately so as to isolate the effect of changing turnover rate without altering pre-therapy viral load. Blue lines, WT virus; red lines, drug-resistant virus; green lines, target cells. These simulations assume a high cost of resistance (*w*_1_ = *k*_1_/*k* = 0.6). Other parameters are as in Table 2 assuming a single population of short-lived infected cells. Interpretation: these simulations illustrate previous theoretical studies showing the concentration of drug-resistant viruses declines transiently following the initiation of therapy.

**Table 1:**
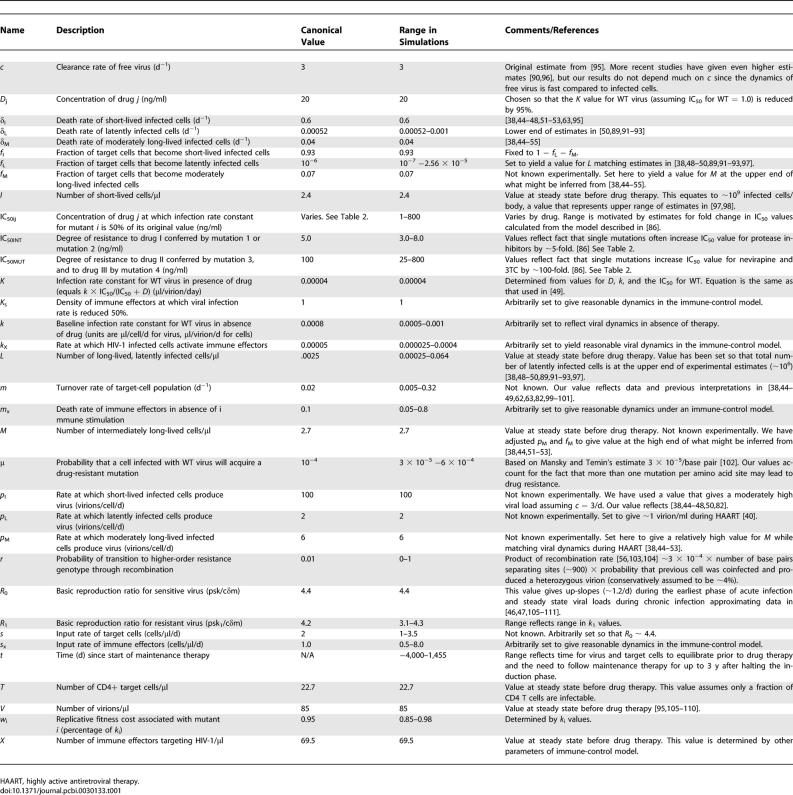
Parameters and Variables Used in the Model

**Table 2:**
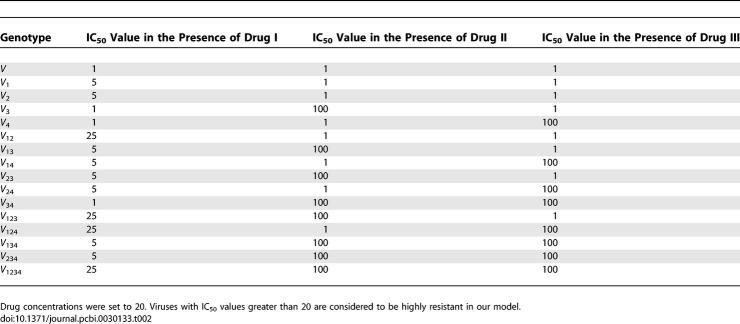
Effects of Individual Drugs on IC_50_ Values

This indicates that the turnover rate of target cells is of major importance in determining the optimal timing of induction therapy relative to the maintenance therapy (as illustrated in Figure 4B), though the replicative fitness of resistant viruses (as quantified by values of *R*_1_ and *R*_0_) also plays a role. Although we have focused on reductions in the infection rate constant as the most logical way of modeling fitness reductions, the dependence of *t*_min_ on *R*_0_ and *R*_1_ indicates that we will observe nearly identical results if the resistant viruses have lower fitness due to a lower burst size or a higher clearance rate.

### Effect of Varying Viral Dynamic Parameters on the Probability of Successful IM Therapy

The results above suggest that induction therapy should be at least 180 d if started at the same time as the maintenance therapy. It also suggests that the optimal time to initiate short-term induction therapy may be several weeks after the start of maintenance therapy. To explore these results in more detail, and to verify that the results are not overly specific to our parameter choices, we systematically varied the key parameters in the full, stochastic model.

We first explored the effect of altering the fitness costs associated with resistance to antiviral drugs (Figure 5A and 5B). As expected, the probability of success decreased with increasing viral fitness under both treatment strategies. Consistent with the equation for *t*_min_ above, the optimal time to intensify therapy increased as the fitness of the resistant virus decreased. Interestingly, we found that changing the fitness of viruses resistant to the induction regimen (drug III) had little or no effect on the optimal time to intensify therapy: the effects depicted in Figure 5B can be ascribed almost entirely to decreased fitness of viruses resistant to the maintenance regimen. As predicted from the equation for *t*_min_ above, we obtained nearly identical results if fitness costs were due to resistant viruses having low burst sizes (unpublished data).

**Figure 5:**
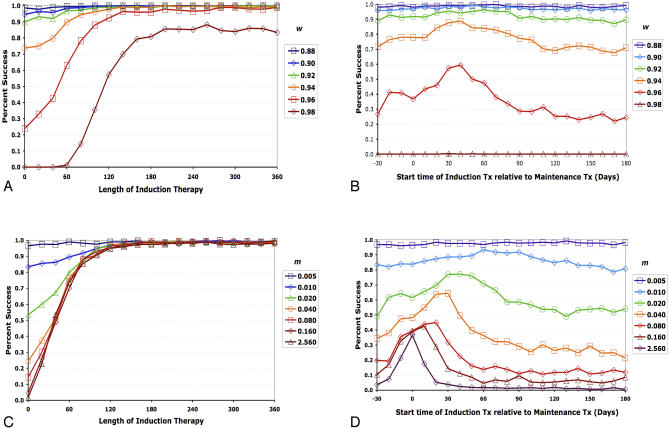
Computer Simulations Demonstrating Success Rates in Eradicating Viruses Resistant to Maintenance Therapy as a Function of Fitness Costs of Resistance and Turnover Rates of Target Cells (A,B) Effects of fitness (*w*) of resistant viruses in the absence of drug. (C,D) Effect of target-cell death rates (*m*) (modeled here with simultaneous increases in *k* in order to keep pre-therapy viral load the same in each simulation). (A) and (C) demonstrate success rates as the duration of induction therapy is increased, and (B) and (D) demonstrate success rates over a range of induction therapy start times. *x*-Axis indicates duration of induction therapy in days (A,C), or the interval between the start of a 30-d induction period and maintenance therapy in days (B,D). Maintenance therapy is assumed to start on day 0. *y*-Axis indicates percentage of simulations in which viral load remained undetectable for at least 3 y after ending induction therapy. Data in each panel were based on 500 replicate simulations. Interpretation: delaying induction therapy until after the start of maintenance therapy results in higher success rates. Under these conditions, starting a 30-d induction period after the start of maintenance therapy usually optimized the probably of success. Success rates decline as the fitness cost of resistance mutations decreases (*w* approaches 1) and as target-cell turnover rates (*m*) increase. The latter effect occurs because target cells necessary for the return of resistant virus rebound more rapidly after therapy at higher turnover rates.

Under simple population genetic models, the frequencies of singly and doubly resistant viruses prior to therapy are proportional to *μ*/*s*, and *μ^2^*/*s^2^*, respectively, where *s* is the selective disadvantage of a drug-resistance mutation [43]. When viruses resistant to the maintenance therapy suffer large fitness costs (e.g., *w_1_* = *w_2_* < 0.65), they rarely, if ever, contribute to the pool of long-lived infected cells. However, when these mutations have very small fitness costs (e.g., *w_1_* = *w_2_* > 0.96), these viruses frequently infect cells destined for latency. (We note that if the cost of resistance to the maintenance therapy is very low, simultaneous triple therapy will fail as well.) We conclude, therefore, that the success of maintenance therapy will depend greatly on resistance mutations having measurable fitness costs.

We next explored the effects of altering the turnover rate (*m*) of the target-cell population, which we accomplished by simultaneously increasing *m* and *k*. From the approximate equation for steady-state viral load:

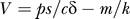
obtained from the simple one-infected cell model, we predict that varying *m* and *k* proportionally will change the dynamics of target-cell renewal without affecting pre-therapy viral load (which is a potentially important confounding factor). In the full model, we found that both the optimal time to intensify therapy and the probability that standard IM therapy is successful increased as target-cell turnover rates decreased (Figure 5C and 5D). Success rates are influenced by *m* because the target-cell populations needed for the growth of resistant viruses recover more slowly when *m* is small. In the simple one-infected cell model, recovery of target cells after therapy is given by

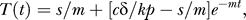
where *t* is time since the initiation of therapy. From this equation, we see that the rate at which target cells return to their pre-therapy steady state is strongly affected by their death rate, *m*.

To examine the role of the latent viral reservoir, we varied the rate at which latently infected cells are created (*f*_L_) in the full, stochastic model. (Unless otherwise specified, all subsequent results are derived from this stochastic model.) With our canonical simulation parameters (with its conservative estimate for the number of latently infected cells), latently infected cells affected outcomes in only a small percentage of cases. The probability of IM therapy failure changed little within the range of *f*_L_ = 10^−8^ – 10^−6^, but decreased significantly for *f*_L_ ≥ 6.4 × 10^−6^ (Figure 6A and 6B, and unpublished data). These results indicate that both IM and conventional triple-drug therapy may fail if the number of latently infected cells is pushed too far above 10^6^, a value near the upper end of experimentally derived estimates (Table 1). As expected from the analytical equations above, altering the number of latently infected cells did not change our previous conclusions concerning optimal timing of IM therapy (Figure 6B).

**Figure 6:**
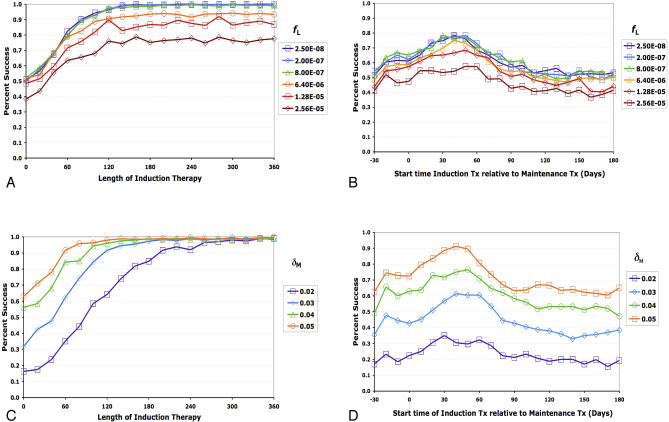
Computer Simulations Showing Relationships Between Long-Lived Infected Cells and Treatment Success Rates (A,B) Effect of proportion of infected cells becoming latently infected quiescent memory T lymphocytes (modeled here by changing *f*_L_). (C,D) Effect of varying the death rate of moderately long-lived infected cells, *δ*_M_ (modeled here with simultaneous increases in *f*_M_ in order to keep the pre-therapy density of moderately long-lived cells the same in each simulation). (A) and (C) demonstrate success rates as the duration of induction therapy is increased, and (B) and (D) demonstrate success rates over a range of induction therapy start times. *x*-Axis indicates duration of induction therapy in days (A,C), or the interval between the start of a 30-d induction period and maintenance therapy in days (B,D). Maintenance therapy is assumed to start on day 0. *y*-Axis indicates percentage of simulations in which viral load remained undetectable for at least 3 y after ending induction therapy. Data in each panel were based on 400 simulations. Interpretation: the death rate of moderately long-lived infected cells is a major determinant of how long induction therapy should last. At expected rates of *f*_L_ (rate at which infected target cells transition to quiescent memory T lymphocytes), success rates depend little on rebound from the latent reservoir. However, success rates decline as the rate of virus input into the latent reservoir exceeds ∼6.4 × 10^−6^ per infected cell, indicating that rebound of resistant virus from the latent reservoir becomes a significant factor.

Finally, we varied the death rate of the moderately long-lived infected cells. In contrast to our conservative estimate for *δ*_L_, our canonical value for the death rate of moderately long-lived cells, *δ*_M_ = 0.04/d, is at the upper end of what might be inferred from second-phase decay rates [38,44–55]. We believe *δ*_M_ = 0.04/d is appropriate because imperfect efficacy and/or poor adherence will cause the second-phase decay rate to be less than *δ*_M_. Second-phase decay rates, furthermore, have been shown to be higher in patients with higher viral loads [55] (the situation modeled here). When we repeated our simulations with lower values for *δ*_M_, we found, as expected, that the duration of induction therapy needed for successful IM therapy increased (Figure 6C). (In these simulations, we simultaneously changed *δ*_M_ and *f*_M_ in order to study the effect of altering *δ*_M_ without affecting the pre-therapy density of moderately long-lived infected cells.) For the case *δ*_M_ = 0.02/d, we observed that induction therapy needed to be at least 300 d to have a high probability of driving viruses resistant to the maintenance therapy to extinction. As expected, changing *δ*_M_ had little effect on the optimal time to intensify therapy (Figure 6D).

### Effect of Resistance Levels and Cross-Resistance on the Probability of Successful IM Therapy

Our canonical simulation includes somewhat arbitrary choices for IC_50_ values for both drug I (for which high-level resistance is assumed to require two mutations) and drugs II and III (for which a single mutation confers high-level resistance). To explore the effects of varying IC_50_ values, we conducted simulations under a range of IC_50_ values for drugs II and III (Figure 7A and 7B) and for drug I (Figure 7C and 7D). As expected, we found that the probability of success in eliminating drug-resistant viruses decreased with increasing IC_50_ values and decreasing drug concentration. As in our previous simulations, the marginal benefit of increasing the length of an induction regimen reached a plateau between 150 d and 270 d. We explored the effect of adding a cross-resistance term wherein resistance to drug II confers partial (or full) resistance to drug III, and vice versa. Success rates decreased with increasing degree of cross-resistance, particularly when induction therapy preceded the start of maintenance (Figure 8A and 8B). However, the qualitative results of our previous simulations remained unchanged.

**Figure 7:**
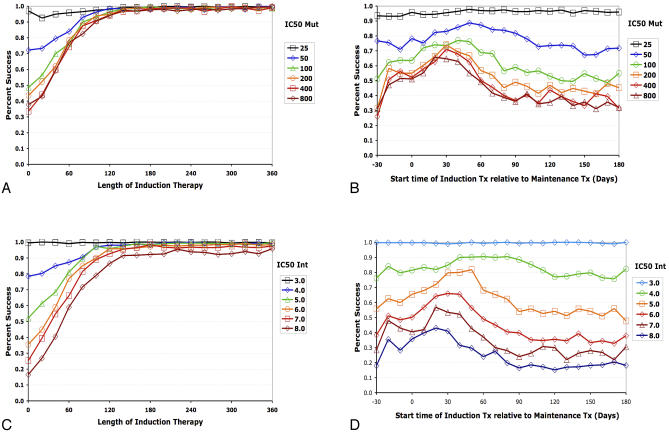
Simulations Demonstrating the Effects of Varying the Degree of Resistance on Treatment Success Rates As in Figures 5 and 6, (A) and (C) demonstrate success rates as the duration of induction therapy is increased, and (B) and (D) demonstrate success rates over a range of induction therapy/therapy intensification start times. IC_50INT_ quantifies the degree of resistance that either mutation 1 or mutation 2 confers to drug I. IC_50MUT_ quantifies both the degree of resistance that mutation 3 confers to drug II and the degree of resistance that mutation 4 confers to drug III. *x*-Axis indicates duration of induction therapy in days (A,C), or interval between start of a 30-d induction therapy and maintenance therapy, in days (B,D). Maintenance therapy is assumed to start on day 0. *y*-Axis indicates percentage of simulations in which viral load remained undetectable for at least 3 y after ending induction therapy. Data in each panel were based on 400 simulations. Interpretation: IM therapy success rates decrease with the degree of resistance conferred by these mutations.

**Figure 8:**
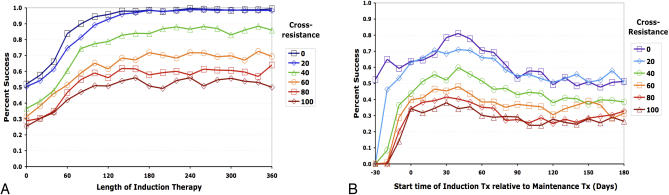
Simulations Demonstrating the Effects of Cross-Resistance on Treatment Success Rates As in Figures 5–7, (A) demonstrates success rates as the duration of induction therapy is increased, and (B) demonstrates success rates over a range of induction therapy/therapy intensification start times. The different lines quantify the degree of resistance that mutations 3 and 4 confer against drugs III and II, respectively. *x*-Axis indicates duration of induction therapy in days (A), or interval between start of a 30-d induction therapy and maintenance therapy, in days (B). Maintenance therapy is assumed to start on day 0. *y*-Axis indicates percentage of simulations in which viral load remained undetectable for at least 3 y after ending induction therapy. Data in each panel were based on 400 simulations. Interpretation: IM therapy success rates decrease with the degree of cross-resistance between mutations 3 and 4.

### Effect of Simultaneously Varying Both the Length and Timing of Delayed-Induction Therapy

All of the delayed-induction therapy simulations above assume a delayed-induction phase of 30 d. To explore the effect of varying the duration and start time of delayed-induction therapy, we repeated our simulations over a range of induction treatment lengths and start times relative to maintenance therapy (Figure 9). For induction therapies of 40 d or less, the optimal time to initiate induction therapy continued to be 30–50 d, as in previous simulations. When the length of induction therapy was increased to 160 d, however, the curve flattened out considerably, indicating that the benefit of delaying induction is diminished at longer treatment durations. This is intuitively reasonable, since longer induction therapies will cover the critical time when resistant viruses are predicted to hit their nadir, even though they might be started well before the optimal therapy intensification times. The benefit of an optimally timed induction therapy, therefore, is most acute when the length of therapy intensification is short.

**Figure 9:**
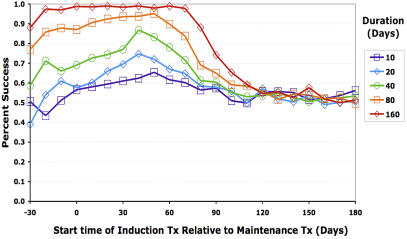
Relationship between Duration of Induction Therapy and Start Time of Induction Therapy Relative to Start of Maintenance Therapy *x*-Axis indicates interval between start of induction and maintenance therapies, in days. Maintenance therapy is assumed to start on day 0. *y*-Axis indicates percentage of simulations in which viral load remained undetectable for at least 3 y after ending induction therapy. Interpretation: the success of IM therapy increases with increasing duration of induction therapy. Delaying the start of induction therapy until ∼40 d after the start of maintenance therapy may be optimal, and the effect of timing is most pronounced with induction therapies lasting 0.5–2 mo. Longer and shorter induction periods are less sensitive to the effects of timing. There is little benefit to adding a delayed-induction therapy at times beyond 90 d after the start of maintenance therapy.

### Effect of Viral Recombination on the Predicted Results of IM Therapy

To explore the effects of viral recombination on these strategies, we extended the model further to account for the effect of recombination between genotypes *V*_12_ and *V*_34_. At realistic recombination rates (i.e., with *r* ≤ 0.01), we observed virtually no effect on the success rate of IM therapy (unpublished data). This is in part because terms of the form *μkTV*_123_, which approximate the rate of production of *V*_1234_ by mutation, are at least an order of magnitude greater than terms of the form *rkI*_12_ *V*_34_, which approximate the rate of input into the *V*_1234_ population by recombination in our model. To achieve a higher-order resistance genotype by recombination, two or more dissimilar resistant virions must coinfect a cell, establish productive infection, and copackage two nonidentical templates to produce a heterozygous virus during virus production. After infection of a new target cell, an odd number of recombination events must occur between templates during reverse transcription, within a locus between the relevant resistance mutations. In the case of drugs targeting protease and reverse transcriptase (the two most common drugs), recombination must occur within a span of ∼900 bp, or roughly one-tenth of the viral genome. Only a fraction of resistant viruses will overcome all of these hurdles. Given published estimates of approximately three recombination events per replication cycle [56], *r* = 0.01 is reasonable, and perhaps somewhat high. To illustrate the ultimate consequences of very high recombination rates, we also performed simulations with unrealistically high recombination rates (i.e., with *r* ≥ 1). At these extreme values, success rates declined in a manner similar to other perturbations that make therapy less likely to be effective (unpublished data). Thus, biologically plausible recombination rates had little qualitative or quantitative effect on the outcomes observed in our four-mutation model.

### Effect of Viral Population Size on the Probability of Eradicating Resistant Viruses

The fact that effective population sizes are so much lower than census sizes is one of the major riddles of HIV-1 evolution. This controversy arises from the observation that the viral effective population size, as measured using standard tools of population genetics, is orders of magnitude lower than the census size (physical count of the number of viruses). In the simulations shown so far, we have conservatively assumed that the dynamics of viral resistance can be described using a model in which the number of viruses in the body equals a liberal estimate of census size. The controversy over viral effective population size has led to suggestions that the use of viral census size is too conservative [57,58]. Unfortunately, it is not clear how to model the effective population size since there is a lack of agreement on why effective population sizes are so low. However, it is possible to explore the effects of some of the more commonly proposed explanations using the modeling framework developed here.

One explanation for low viral effective population size is that most of the infected cells and virions assayed by PCR are noninfectious. If this were the entire explanation for extremely low effective population sizes, use of current estimates of census size would be inappropriate. To explore what occurs if very few virions and integrated proviruses are replication-competent, we repeated our simulations with a census size 10,000-fold lower than the one used previously. Under this assumption, we obtained qualitatively similar results under a treatment regimen in which both the induction and the maintenance therapies consist of one drug. While a reduced therapy burden would be a welcome finding, two-drug therapies have not been generally successful, suggesting that these conditions are a less accurate approximation of biological conditions.

Another possibility is that the effects of a genetic bottleneck during primary infection and rapid turnover of viral populations due to strong immune selection periodically purge HIV-1 populations of genetic variation. Because the effective population size is proportional to the amount of genetic variation, these factors would have a large negative effect on the measured effective population size during primary infection. To examine the impact of these processes on the dynamics of resistant virus, we set viral load to a very low value at the beginning of primary infection, and simulated immune selection for a character unrelated to resistance mutations, starting near day 200. We found that neither mechanism for low effective population size had a significant long-term impact on the frequencies of drug-resistant viruses (unpublished data). Although these simulations cover only some of the possible mechanisms for low effective population size [59–61], they indicate that it is possible to appropriately model drug therapy using population sizes similar to the census size, regardless of the calculated effective population size.

### Behavior of Drug-Resistant Viruses under an Immune-Control Model

The results above are all based on a “standard” model that assumes that HIV is limited in vivo by the supply of CD4^+^ target cells [38,45–48,62,63]. We have chosen to use this standard model because it is supported by independent lines of evidence [64] and is well-studied mathematically, and because there is no clear consensus on appropriate methods to model immune responses. However, some modelers have argued that viral load is determined primarily by the dynamics of the immune response (reviewed in [65]). To verify that our results are not specific to this target-cell limited model, we have performed analogous simulations under a model in which viral populations are limited instead by the immune effectors (ones that act by preventing virus from infecting cells). In Figure 10A, we show using this model that drug-resistant viruses transiently drop in density following drug therapy in a manner very similar to that which occurred under the one-drug, one-cell, one-mutation, target-cell limited model in Figure 4A. When this model was extended to account for moderately long and very long-lived infected cells and varying turnover rates for immune effectors, we obtained results analogous to those for the stochastic target-cell limited model (Figure 10B). In both models, the essential feature is that the environment for resistant viruses improves as viral load decreases, and in both models the length of the dip depends on how rapidly “the environment” improves. In the target-cell limited model, drug-resistant viruses showed a larger transient reduction if target cells regenerated slowly after therapy. In the immune-control model, drug-resistant viruses underwent larger transient declines if the HIV-specific effector cells decayed slowly during drug therapy.

**Figure 10:**
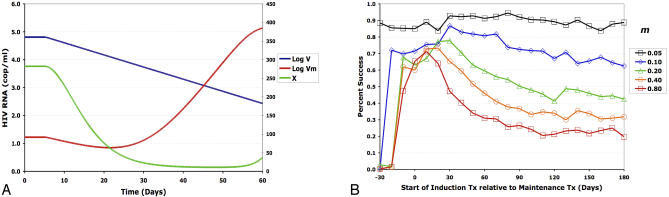
Computer Simulations of Dynamics of Drug-Resistant Virus under Simple Immune-Control Model (A) Immune-control analog of the one-cell, one-drug model presented in Figure 4. (B) Effect of changing turnover rate of immune effectors under the immune-control analog of the full model explored in Figures 5–9. In this simulation, the turnover rate of the immune effectors was modeled by simultaneously increasing *s*_X_, *m*_X_, and *k*_X_. Here, *k* = 0.00085, *T* = 1,000, *μ* = 6 × 10^−4^, and *w*_1_ = *w*_2_ = *w*_3_ = *w*_4_ = 0.9. Other parameters are as in Table 2. Interpretation: changing the factor responsible for controlling viral load did not change the conclusion that drug resistant viruses will decrease transient after drug therapy. As with the target-cell limited model, the rate at which the factor that controlled viral load changed after therapy played a major role in determining when therapy should be intensified.

## Discussion

In this study, we have used a detailed differential equation model to investigate induction–maintenance (IM) strategies for treating HIV-1 infections. In these strategies, an induction regimen is used to drive viral load to low levels before switching patients to a simpler and potentially better tolerated long-term maintenance regimen. We find that an appropriately deisgned IM regimen is likely to result in long-term suppression of viremia, and may also result in the eradication of minority virus populations resistant to the maintenance regimen. The marginal benefit of increasing the induction phase starts to level off between 4 and 10 mo, depending on the parameter choices. Interestingly, we find that in cases where target-cell populations recover slowly after ART, the optimal time to initiate a short-term induction regimen may be optimally started several days to weeks after the start of maintenance drugs. (This *delayed*-induction therapy may also be referred to as delayed-intensification or booster therapy.) These delays are advantageous because viruses resistant to the maintenance regimen briefly decline after exposure to the maintenance drug, due to reduced mutational input from the majority sensitive population. These resistant viruses do not increase again until the environment for the virus improves (modeled here as a recovery in target-cell populations). Intensifying therapy when the resistant virus population is close to its nadir maximizes the effectiveness of the additional therapy. These results therefore illustrate the importance of considering dynamic feedback mechanisms such as those that occur under classical predator–prey models in ecology [66,67] when implementing IM regimens.

Although our exploration of this model has caused us to view IM therapy in an optimistic light, our model predicts that IM therapies can fail under a variety of conditions, including situations in which drug resistance imposes little or no fitness costs (Figure 5A and 5B), situations in which latently infected cells are formed at high rates (Figure 6A and 6B), and situations in which the primary mutations responsible for drug resistance have large effects on the IC_50_ values, either directly (Figure 7) or indirectly via cross-resistance (Figure 8A and 8B). Our specific predictions about the optimal length for the induction period, likewise, depend on the size of the overall viral reservoir and the rate of the decay of moderately long-lived infected cells (the primary determinant of optimal induction length). Finally, as discussed above, our finding that the best time to intensify therapy is often several days to weeks after the start of regular therapy depends critically on two parameters: the fitness of the resistant virus and the rate at which target-cell populations recover after initiation of therapy. The lower the fitness of resistant viruses and the slower the rate of recovery of target cells (or other factors regulating viral density), the later the optimal time to maximize therapy. In cases where target-cell populations increase rapidly, or when other factors that limit viral replication decay quickly during therapy, delaying the induction phase may not be beneficial.

These findings may be important in several clinical scenarios. IM therapy may be useful in resource-poor settings where patients have limited access to antiretroviral drugs. In these settings, it is particularly important to minimize the chance of selecting for drug-resistant viruses during the initial attempt to administer antiretroviral drugs. In addition, an intensification–maintenance approach could provide protection against the development of drug resistance in antiretroviral-naive patients, particularly in patients infected by a donor with known poor adherence to medications (in which case it would be advisable to consider a maintenance phase consisting of three or more drugs, as opposed to the two-drug maintenance regimens modeled here). Recent estimates suggest that up to 10%–15% of treatment-naive patients harbor one or more drug-resistance mutations [68–70], and this problem is likely to increase with increasing availability of ART. Finally, the principle of IM approaches could also be applied to the difficult problem of salvage therapy. The latter two scenarios have not been specifically modeled here.

The results presented here must be weighed against several practical considerations: a two-drug maintenance regimen may incur a higher failure risk among patients prone to subtherapeutic drug levels for any reason, since there will be a reduced level of concurrent coverage by other agents in the regimen. It is also essential that the maintenance regimen not include drugs for which the patient previously developed drug resistance, a requirement that is complicated by the problem of cross-resistance. In addition, it would be highly desirable that agents used in maintenance therapy be simple and well-tolerated, with favorable pharmacokinetics, and have a high barrier to the development of resistance—both in terms of the number of mutations required for resistance and the fitness of the resulting mutants. By contrast, the requirements for induction regimens are considerably less stringent: induction therapy must be able to suppress replication of viruses resistant to the maintenance regimen and be free of intolerable adverse effects during short-term use.

Although we have gone to considerable lengths to make the model realistic, we still make a number of simplifying assumptions. First, we ignore drug redistribution, and assume that drug levels immediately reach the therapeutic window at the time of initiation, remain constant during therapy, and fall to zero at discontinuation. There will clearly be some deviation from these ideal conditions in vivo because of pharmacokinetic “loading effects,” individual failure to adhere to treatments, antagonistic drug interactions, and other factors. Although we believe that four mutations are sufficient to capture the basic behavior of drug resistance, this is clearly a simplification, as are some of our assumptions about IC_50_ values and cross-resistance. Our point is to make a reasonable model that captures key features, not to make a complete model of drug resistance. We have also neglected reversion of drug-resistant variants to WT virus. However, this effect is likely to be small under drug therapy, and would result in lower failure rates than modeled here.

In building our model, we assumed that double therapy usually fails and that triple therapy usually succeeds, as has been observed in clinical practice. There are, of course, wide regions of parameter space where double therapy always succeeds and, conversely, where triple therapy always fails, and it is possible that many real patients could fall into one of these two categories. Although the specific simulations presented here would not be relevant to these patients, the same concept (but with a different number of drugs) can be applied to these patients. The key to applying IM strategies to such patients would be develop methods for distinguishing among patients whose maintenance therapies would require one, two, three, or more drugs.

Finally, our model assumes a degree of fitness cost of resistance to drugs. Several studies have linked the presence of resistance mutations with decreased RT processivity [71], reduced replicative capacity in vitro [72–75], a competitive disadvantage against WT viruses in competition assays [75], lower viral loads, and lower rates of CD4 T cell loss in vivo [72,73,75], and have shown a tendency for overgrowth by WT viruses after discontinuation of therapy in cases of mixed infection [76,77]. As shown in Figure 5A and 5B, the probability of treatment success drops dramatically as the cost of resistance decreases. An essential feature of any two-drug maintenance regimen, therefore, is that the maintenance regimen includes drugs for which resistant mutations incur measurable fitness costs. In cases where fitness costs are small, it would be advisable to choose maintenance regimens in which four or more mutations are required for resistance (something that can easily be implemented using a three-drug maintenance regimen).

Key experiments needed to test the model's assumptions would focus on how the concentration of resistant viruses residing in short-lived, moderately long-lived, and latently infected cells changes during the first 90 d of therapy. Experiments designed to test the prediction that resistant viruses decrease transiently during therapy could be particularly informative. A better understanding of factors that allow for continued replication in the face of various therapies (e.g., identification of sanctuary sites in which drugs do not penetrate) would also be very important. More generally, experiments designed to improve our understanding of viral effective populations size and factors that control viral load in the absence of therapy could lead to the construction of more realistic models for viral dynamics. Also, since our model shows that the probability of therapy success decreases as the number of latently infected cells increases, our study suggests that it would be useful to obtain additional quantitative estimates of the size of the latent viral reservoirs. Most studies of the latent reservoir have focused on blood. If less intensively studied sites such as the lung, brain, or gastrointestinal tract were found to have larger than expected numbers of latently infected cells, it might be necessary to choose more conservative treatment strategies.

In addition to HIV-1, IM approaches are being used for the treatment of a growing number of infectious illnesses, including active tuberculosis [78], bacterial endocarditis [79], and prosthetic joint infections [80], and have widespread application in oncology. In these settings, induction therapy is usually timed to coincide with initiation of maintenance therapy, and maintained for an empirically determined period of time. Although the replication dynamics of the pathogenic elements in these cases (i.e., infecting microorganisms or aberrant host cells) differ significantly from those of HIV, the chronic nature of these conditions, the requirement for long-term therapy, and the potential for developing resistance to drugs and immune responses pose similar challenges to the host. The counterintuitive results that have emerged from our analysis of HIV replication under therapy suggest that it may be beneficial to explore dynamic modeling approaches in these cases as well.

## Materials and Methods

### Overview of the model-building process.

As with most biological models, certain parameters and assumptions are better supported than others. Parameters used in our model are given in Table 1. These values resulted from a sequential process in which we first fixed parameters, such as viral load, *δ*_I_, *δ*_M_, and *δ*, which have been characterized experimentally. We then manipulated unknown/less-well–characterized parameters to match in vivo data on the viral kinetics during primary infection, during therapy, and after a treatment interruption. Most of these parameters were set to yield conservative (i.e., higher than average) estimates for the number of infected cells. We then varied the drug concentrations and IC_50_ values (within estimated ranges) to match experimental observations that triple therapy is usually successful but double therapy usually fails. After completing these three steps, we performed our key exploratory simulations in which we examined the effects of varying the length and timing of induction therapy. Simulations were repeated across a wide range of reasonable values for parameters that remain poorly characterized by experimental methods (e.g., target-cell turnover rates).

### Equations for viral dynamics.

Dynamics of infection were simulated using an extension of a commonly used model for viral dynamics [38,41,45–48,62,81–85] that assumes that viral load is limited by the supply of CD4^+^ target cells. Our model consists of 65 differential equations accounting for target cells, free virions, three types of infected cells, and 16 viral genotypes (Figure 1B). The dynamics of target cells and drug sensitive viruses are given by

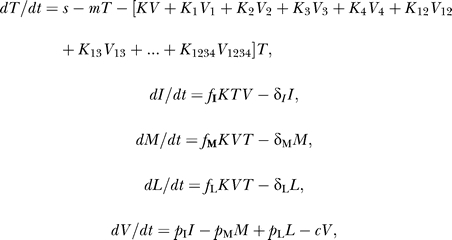
where *I*, *M*, and *L* represent short-lived, moderately long-lived, and latently infected cells, respectively; *V* represents free virions; *T* represents target cells; *f_M_* and *f*_L_ are the fractions of target infected cells that become moderately long-lived and latently infected cells upon HIV-1 infection; *f**_I_*** = *1* − *f**_M_*** − *f_L_*; *s* is the input rate of target cells; *m* is the death rate of target cells; *δ*_I_, *δ*_M_, and *δ*_L_ are the death rates of short-lived, moderately long-lived, and latently infected cells, respectively; *p_I_*, *p_M_*, and *p_L_* are the rates at which short-lived, moderately long-lived, and latently infected cells produce virus; *c* is the clearance rate of free virus; *t* is time in days; *K* is the rate at which WT virus infects cells, and *K_i_* is the rate at which virus with resistance mutation *i* infects target cells in the presence of therapy. To model the effects of drugs on these different viruses, we assume that infection rate constants *K*, *K*_1_, *K*_2_, ..., *K*_1234_ decline in the presence of drug-using functions described below (see Modeling of viral replication under drug therapy).

The dynamics of mutants partially resistant to drug I, but sensitive to drugs II and III, are given by equations of the form:

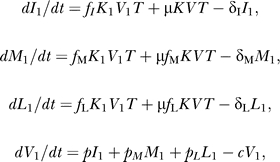
where *μ* is the probability that a cell infected with WT virus will acquire a resistance mutation to one of these drugs. The equations of other resistant mutants are straightforward extensions of these equations with sequential mutation accumulation. For example, the dynamics of mutants with high-level resistance to drug I, but sensitive to drugs II and III, are given by the equations.

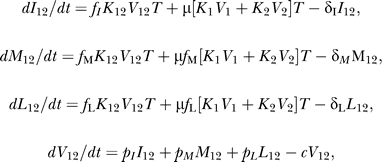
while the dynamics of mutants resistant to all four drugs is given by the equations

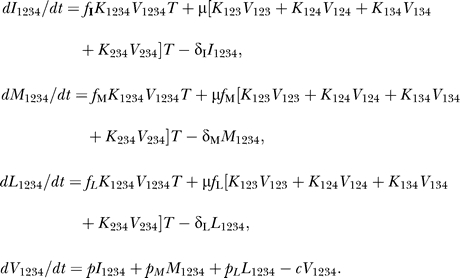



We note that this model assumes that reverse mutations from resistance to sensitivity is negligible. Another cryptic assumption is that short-lived, long-lived, and latently infected cells are derived from a single population of CD4^+^ target cells, as modeled by Nowak et al. [41]. In preliminary simulations and/or calculations, we have determined under reasonable conditions that neither of these factors has much effect on our qualitative conclusions.

### Extinction conditions.

The extinction threshold was set to 3 × 10^−9^ infected cells/μl, which is roughly equivalent to one infected cell per 2 × 10^11^ CD4 cells (the approximate total body CD4 cell population). In preliminary work, we found that it is almost impossible to eliminate viruses resistant to any single drug during triple-drug therapy. IM therapy was therefore considered to be successful when the concentration of viruses and cells infected with viruses resistant to both of the drugs in a two-drug maintenance regimen fell to zero or if viral load failed to rebound for a period of 3 y after ending induction therapy.

### Modeling of viral replication under drug therapy.

To allow for imperfect drug efficacy against WT virus, we assumed that the infection rate constant for genotype *i* in the presence of drug *j* can be modeled as:

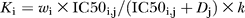
where *k* is the baseline infection rate constant for WT virus in the absence of drug, *w*_i_ is the replicative fitness cost associated with mutation *i* (expressed as a percentage of *k*), IC50_i,j_ is the concentration of drug *j* at which infection rate constant for mutant *i* is 50% of its original value, and *D_j_* is the concentration of drug *j* [49]. In our four-mutation system, mutations 1 and 2 confer partial resistance to drug I, while mutations 3 and 4 confer substantial (though not 100%) resistance to drugs II and III, respectively. For the “canonical case,” we assumed that mutations 1 and 2 each confer a 5-fold increase in IC_50_ value against drug I, resulting in a 25-fold increase in resistance for the double mutant *V*_12_ as expected [86], while mutations 3 and 4 confer 100-fold increases in IC_50_ values against drugs II and III, respectively. In the figures, we refer to the fold increase in resistance conferred by mutations 1 or 2 as “IC50INT” (since these mutations confer an intermediate level of resistance), and the fold increase in resistance conferred by mutations 3 and 4 as “IC50MUT” (since these mutations confer high-level resistance; i.e., they are completely mutated). Under this model, resistance to drug I would be analogous to resistance to a protease inhibitor, while resistance to drugs II and III would resemble resistance to nucleoside reverse transcriptase inhibitors and first-generation nonnucleoside reverse transcriptase inhibitors. The resulting IC_50_ values are summarized in Table 2. To calculate the infection constants in the presence of multiple drugs, we used generalizations of the IC_50_ formulas given above, wherein fitness effects and IC_50_ effects are multiplied together to give the composite infection rate constant. For example, the infection rate constant for the quadruple mutant V_1234_ in the presence of drugs is given by:

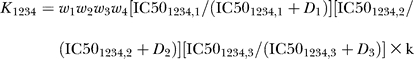
where *k* is the baseline infection rate constant for WT virus in the absence of drug; *w*_1_, *w*_2_, *w*_3_, and *w*_4_ are the negative fitness effects associated with each resistance mutation; IC50_1,1_, IC50_1,2_, and IC50_1,3_ are the IC_50_ values for genotype V_1_ against drugs I, II, and III, respectively; IC50_1234,1_, IC50_1234,2_, and IC50_1234,3_ are the IC_50_ values for genotype V_1234_ against drugs I, II, and III, respectively; and *D*_1_, *D*_2_, and *D*_3_ are the concentrations of drugs I, II, and III, respectively. In the presence of drug, we assumed drug concentration values of 20 ng/ml. In our model, drug concentrations immediately rise to therapeutic levels or fall to zero when therapy is changed. In preliminary calculations, we have determined that pharmacokinetic transients have relatively little effect on our qualitative results under reasonable conditions.

Finally, we modeled (reciprocal) cross-resistance between mutations conferring resistance to drugs II and III by setting the IC_50_ value each of these drugs to IC50_WT_ × (IC50_MUT_ / IC50_WT_)^α^, where *α* is a coefficient giving the degree of cross-resistance. When *α* = 0, the IC_50_ value equals that of the WT value; when *α* = 1, the IC_50_ value of the mutant equals that of the mutant that is resistant to the other drug. These *α* values were then converted to percentages, where 0% indicates no cross-resistance, and 100% indicates that mutations conferring resistance to drug II are equally resistant to drug III and vice versa.

### Modeling the effects of recombination.

In models with three mutations, recombination acts only on the same order as the mutation rate, since the triple mutant *V*_123_ can be created by either one mutation added to *V*_12_ or recombination between *V*_12_ and *V*_3_. However, in models with four or more mutations, recombination between *V*_12_ and *V*_34_ reduces the number of mutation/recombination events needed to create a fully resistant virus. To account for recombination without adding a huge number of terms, we assumed that infection of *I*_34_ by *V*_12_ or infection of *I*_12_ by *V*_34_ results in the formation of the quadruple mutant with probability *r*, where 0 ≤ *r* ≤ 1. For example, the equation for short-lived infected cells with virus with all four resistance mutations becomes:

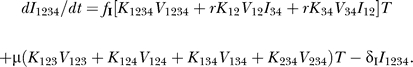



Modifications for *M*_1234_ and *L*_1234_ were similar.

### Stochastic effects at low population densities.

To account for random genetic drift occurring at low population densities, we used stochastic terms similar to those used in [46] to model populations near the cutoff for extinction. For each time-dependent variable *x* (e.g., *I*, *V*), we first determined if *x* < *n_s_x_min_*, where *n_s_* is the number of copies below which *x* is subject to stochastic forces and *x_min_* is the concentration at which there is only one virus or infected cell in the body. For *x* ≥ *n_s_x_min_*, we set *x*(*t* + *h*) = *x*(*t*) + [*B*(*x*) − *M*(*x*)]*h*, where *h* is the step size, *B*(*x*) is the sum of the “birth” terms, and *M*(*x*) is the sum of the “mortality” terms. For *x* < *n_s_x_min_*, we set *x*(*t* + *h*) to *x*(*t*) − 1, *x*(*t*), or *x*(*t*) + 1 according to the probabilities *hM*(*x*), 1 − [*M*(*x*) *+ B*(*x*)]*h*, and *hB*(*x*). Use of deterministic equations for *x* > *n_s_x_min_* strikes a balance between the need to account for stochastic effects at low population densities and the need to reduce computation times at higher densities where stochastic effects are negligible. Since preliminary runs with *n_s_* = 25, 50, 100, and 200 gave similar results (but clearly distinct from *n_s_* = 1 or *n_s_* = 5), we reasoned that *n_s_* = 25 would be sufficient to capture most of the stochastic variation that occurs at low density. Probabilities were determined using the random number generator MT19937 [87]. Simulations performed using the random number generator ran2 [88] yielded indistinguishable results (unpublished data).

### Starting parameter values.

To create a realistic simulation of IM therapy, we adjusted the parameters to match the dynamics of viral decay during potent combination therapies [38,40,89,90]. Prior to the initiation of therapy, we assumed that there are ∼10^10^ viruses, ∼3 × 10^8^ short-lived infected cells, ∼10^7^ moderately long-lived infected cells, and ∼10^6^ latently infected cells per body. Unless otherwise stated, other parameter values used were: *s* = 2.0 cells/d, *m* = 0.02 cells/d, *k* = 0.0008 cells × μl/d, *w*_1_ = 0.95, *w*_2_ = 0.95, *w*_3_ = 0.95, *w*_4_ = 0.95, *δ*_I_ = 0.6 cells/d, *δ_M_* = 0.04 cells/d, *δ_L_* = 0.00052 cells/d, *f*_M_ = 0.07, *f_L_* = 10^−6^, *p* = 100 virions/d, *p*_M_ = 6 virions/d, *p*_L_ = 2 virions/d, *c* = 3 d^−1^, and *μ* = 1 × 10^−4^. All three drugs (*D*_1_, *D*_2_, *D*_3_) are set at 20 ng/ml when these drugs are present. The input rate of target cells, *s*, was set so that the steady state concentration of target cells is 100 cells/μl, or approximately 10% of a typical peripheral blood CD4 T cell count, since not all CD4^+^ T cells are susceptible to HIV-1 infection. Units for target cells are based on a total estimate of 2 × 10^11^ CD4 cells per body, of which 2% are in blood. The stochastic cutoff threshold was set at one infected cell per body, or 3 × 10^−9^ cells/μl. The death rate of latently infected cells of *δ_L_* = 0.00052/d (*t*_1/2_ = ∼44 mo) was conservatively set to one of the lower experimental estimates [50,89,91–93]. The mutation rate was deliberately set to approximately three times the estimated per-base rate to account for the fact that more than one nucleotide mutation may lead to an amino acid change that results in resistance. In all simulations, we assume that fitness effects are multiplicative: that is, that *k*_12_ = *k*_1_ *k*_2_ / *k*, *k*_13_ = *k*_1_ *k*_3_ / *k*, *k*_23_ = *k*_2_ *k*_3_ / *k*, and *k*_123_ = *k*_1_ *k*_2_ *k*_3_ / *k* ^2^, as in [94]. The effects of changing less well-quantified parameters, such as *m* and *k*, are summarized in the results.

### Immune-control model.

Although we focus on the target-cell limited model described above, we also explored a simple immune-control model to determine how dependent our qualitative results are on the factors that regulate HIV-1 density. In our immune-control model, the virus population expands exponentially without limitation in the absence of immunity. Immune effectors, which increase at a rate proportional to the number of infected cells, interfere with the ability of virus to infect cells (as might happen if immune cells release chemokines and/or neutralizing antibodies). We implemented this initially using the following model with one mutation and one type of infected cell:

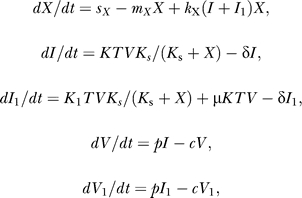
where *X* is the concentration of immune effectors, *s*_X_ is the rate of appearance of immune effectors in the absence of immune stimulation, *m*_X_ is the death rate of immune effectors, *k*_X_ is the rate at which HIV-1–infected cells activate immune effectors, and *K_s_* is a saturation constant describing the negative effect that the immune effectors have on the ability of HIV-1 to initiate infections. The symbols *T*, *I*, *I*_1_, *V*, *V*_1_, *K*_1_, *p*, *c*, *δ*, and *μ* have the same meanings as in the target-cell limited model above, though when simulating dynamics under this model, we assume that *T* does *not* change over time. To extend this immune-control mechanism to the full, stochastic model, we made analogous extensions, setting *dX* / *dt* = *s*_X_ − *m*_X_ *X* + *k*_X_(*I* + *I*_1_ + … + *I*_1234_ + *M* + … + *M*_1234_)*X* and multiplying the infection rate constants (*K*, *K*_1_, *K*_2_, *…, K*_1234_) by *K*_s_ / (*K*_s_ + *X*), while keeping *T* constant. To simulate drug treatment for different rates of turnover of immune effectors without also changing pretherapy viral loads, we increased *s*_X_, *m*_X_, and *k*_X_ proportionately. (The latter is needed since steady-state viral load is the sum of terms proportional to *s*_X_ / *k*_X_ and *m*_X_ / *k*_X_.)

## Abbreviations

ART: antiretroviral therapy
IM: induction–maintenance
WT: wild-type
